# Drugs and convalescent plasma therapy for COVID-19: a survey of the interventional clinical studies in Italy after 1 year of pandemic

**DOI:** 10.1186/s13063-022-06474-8

**Published:** 2022-06-22

**Authors:** Maria Puopolo, Cristina Morciano, Maria Buoncervello, Chiara De Nuccio, Rosa Luisa Potenza, Elena Toschi, Lucia Palmisano

**Affiliations:** 1 Department of Neuroscience, Istituto Superiore di Sanità, Rome, Italy; 2 Research Coordination and Support Service, Istituto Superiore di Sanità, Rome, Italy; 3 National Center for Drug Research and Evaluation, Istituto Superiore di Sanità, Rome, Italy

**Keywords:** COVID-19, Clinical trials, Drugs, Convalescent plasma, Therapeutic class, Study design

## Abstract

**Background:**

The 2019 novel coronavirus disease (COVID-19) pandemic has highlighted the importance of health research and fostered clinical research as never before. A huge number of clinical trials for potential COVID-19 interventions have been launched worldwide. Therefore, the effort of monitoring and characterizing the ongoing research portfolio of COVID-19 clinical trials has become crucial in order to fill evidence gaps that can arise, define research priorities and methodological issues, and eventually, formulate valuable recommendations for investigators and sponsors. The main purpose of the present work was to analyze the landscape of COVID-19 clinical research in Italy, by mapping and describing the characteristics of planned clinical trials investigating the role of drugs and convalescent plasma for treatment or prevention of COVID-19 disease.

**Methods:**

During an 11-month period between May 2020 and April 2021, we performed a survey of the Italian COVID-19 clinical trials on therapeutic and prophylactic drugs and convalescent plasma. Clinical trials registered in the Italian Medicines Agency (AIFA) and ClinicalTrials.gov websites were regularly monitored. In the present paper, we report an analysis of study design characteristics and other trial features at 6 April 2021.

**Results:**

Ninety-four clinical trials planned to be carried out in Italy were identified. Almost all of them (91%) had a therapeutic purpose; as for the study design, the majority of them adopted a parallel group (74%) and randomized (76%) design. Few of them were blinded (33%). Eight multiarm studies were identified, and two of them were multinational platform trials. Many therapeutic strategies were investigated, mostly following a drug repositioning therapeutic approach.

**Conclusions:**

Our study describes the characteristics of COVID-19 clinical trials planned to be carried out in Italy over about 1 year of pandemic emergency.

High level quality clinical trials were identified, although some weaknesses in study design and replications of experimental interventions were observed, particularly in the early phase of the pandemic.

Our findings provide a critical view of the clinical research strategies adopted for COVID-19 in Italy during the early phase of the pandemic. Further actions could include monitoring and follow-up of trial results and publications and focus on non-pharmacological research areas.

**Supplementary Information:**

The online version contains supplementary material available at 10.1186/s13063-022-06474-8.

## Introduction

The emerging SARS-CoV-2 coronavirus disease, known as COVID-19, has rapidly developed into a pandemic with a disruptive impact on health and economy. The clinical research community worldwide has responded to the pandemic at an unprecedented speed to produce evidence to prevent, diagnose, and treat COVID-19. A massive number of clinical trials have been registered at the various dedicated web registries, and as the number of clinical trials has increased, the initiatives to map the clinical research landscape and its evolution over time have increased too [1–6].

Several analyses of the characteristics of the registered COVID-19 clinical trials have already been conducted [7–9], showing redundancy and methodological issues [7], thus highlighting the need for a better coordination and collaboration of the research effort. Indeed, monitoring ongoing clinical research can be crucial for the management of COVID-19 emergency since it offers information that may improve research coordination across research entities, both within and among countries. This would avoid unnecessary duplication and fragmentation of the efforts [10] and offer the opportunity to share knowledge on methodology at the planning stage of the trial with substantial improvement of the quality of the evidence [11].

In Italy, the Italian Medicine Agency (AIFA) was entrusted with the task of managing the submission and authorization process of all clinical trials on drugs for the treatment and prevention of COVID-19. Specifically, according to the “Cura Italia” Decree Art. 17 [12], all clinical trial protocols on medicinal products for human use and medical devices for patients affected by COVID-19 had to be evaluated by the Technical Scientific Committee (CTS) of AIFA and by the Ethics Committee of the National Institute for Infectious Diseases (INMI) Lazzaro Spallanzani (single National Ethics Committee for COVID-19 clinical studies). In compliance with such measure, the list of authorized pharmacological COVID-19 clinical trials and related study protocols are made available in the AIFA website, whereas clinical trials of non-pharmacological interventions (such as infusion of plasma from convalescent COVID-19 patients) as well as pharmacological study proposals still under evaluation or rejected are not present in the website.

The aims of the measures introduced with the “Cura Italia” decree were to speed up the approval process, guarantee a highly qualified assessment, ensure the transparency of the approval process, and at the same time, facilitate the exchange of information within the scientific community [13].

In this context, the Working Group “Clinical Trials” of the Italian National Institute of Health [Istituto Superiore di Sanità (ISS)] has regularly mapped interventional trials on medicinal products and convalescent plasma planned in Italy, by integrating information from the AIFA website and the ClinicalTrials.gov website. Nine infographics were produced and published between 29 May 2020 and 22 April 2021. The main objective was to disseminate useful information to researchers, health professionals, funders, policy makers, and citizens. Here, we report the results of the mapping analysis of clinical research on COVID-19 in Italy updated to about 1 year after the start of the pandemic officially declared by WHO on 11 March 2020 [14].

## Methods

### Study types and data sources

We identified and analyzed the preventive and therapeutic COVID-19 interventional clinical studies with drugs and plasma from convalescent patients planned in Italy at about 1 year from the beginning of the pandemic emergency [14].

To perform this survey, we used two data sources. The main source was the open-access AIFA website specifically established for COVID-19 clinical studies [15] in compliance with the Decree Law “Cura Italia” providing extraordinary measures to face the medical emergency (published on 18 March 2020) [12]. The list of pharmacological COVID-19 clinical trials evaluated and fully approved by either the CTS of AIFA and the National Unique Ethic Committee of the INMI Lazzaro Spallanzani—regularly updated—is available on the AIFA website. The related study protocols are also accessible on the same website. The second data source was ClinicalTrials.gov website, the portal for globally conducted clinical trials of the National Institutes of Health—US National Library of Medicine [16], which made available the list of registered COVID-19 clinical trials with related study information.

### Data collection and analysis

Data on COVID-19 clinical trials approved by AIFA were obtained through the periodic consultation of the dedicated website [15] and the download of the related study protocols. The following study information has been extracted: identification number (EudraCT Number), title, acronym, primary purpose (treatment, prevention), experimental intervention, phase, design (single group, parallel groups, cross-over, sequential), randomization (yes/no), masking (yes/no), multicentric study (yes/no), international study (yes/no), planned sample size, and sponsor type (Industry/No industry). All the collected information was included into an ad hoc established form. Data were updated to 6 April 2021.

The list of COVID-19 clinical trials registered on ClinicalTrials.gov portal [17] visited on 6 April 2021 was used. The list was obtained through the search term “COVID-19,” and the search synonyms “COVID,” “SARS-CoV-2,” “severe acute respiratory syndrome coronavirus 2,” “2019-nCoV,” “2019 novel coronavirus,” and “Wuhan coronavirus.” Clinical trials were extracted by applying the filter “Interventional (Clinical Trial)” in the field “Study Type.” The information data of selected studies were downloaded in “Comma-separated values” format by including “all available columns.”

By using the downloaded dataset, Italian studies have been identified by the presence of the “Italy” string in the “location” column. Whenever the location information was missing, Italian studies were identified by looking at data reported in “sponsor/collaborators” or “study title” or “principal investigator” columns. Clinical studies investigating “medical device,” “diagnostic test,” “behavioural interventions,” “procedure,” “radiation,” “dietary supplement,” and “other” were excluded. Study data (see list described above for studies in the AIFA website) were derived from the downloaded information. Studies were identified through their assigned NCT number.

Any ambiguity detected during the categorization of the available information, such as the classification of the characteristics of the study design or experimental intervention, was collegially discussed and a decision was taken by common consent.

A procedure was developed to integrate the two data sources: the list of Italian clinical trials identified using the ClinicalTrials.gov registry was compared with that presented in the AIFA website in order to detect any eventual duplicates. In case of duplicates, the information reported in the AIFA website was considered.

The pharmacological interventions were classified by using the DrugBank database linked to the browser The Anatomical Therapeutical Chemical Classification System (ATC), which allows to get detailed information on the drugs and their specific targets [18].

Descriptive analyses of the characteristics of the study designs (overall and through data stratification by source) have been done by calculating frequencies and relative frequencies for categorical/categorized variables, and medians with interquartile range for continuous variables. To this aim, the sample size was categorized as 1-100, 101-1000, >1000. Furthermore, the experimental interventions have been listed and summarized by therapeutic classes and subclasses.

Multiarm trials were listed and platform trials—which investigate multiple treatments simultaneously with the aim of finding the best treatment for a disease [19]—were extracted.

The statistical software STATA 16 was used.

## Results

### Collection of Italian clinical trials

**Fig. 1:**
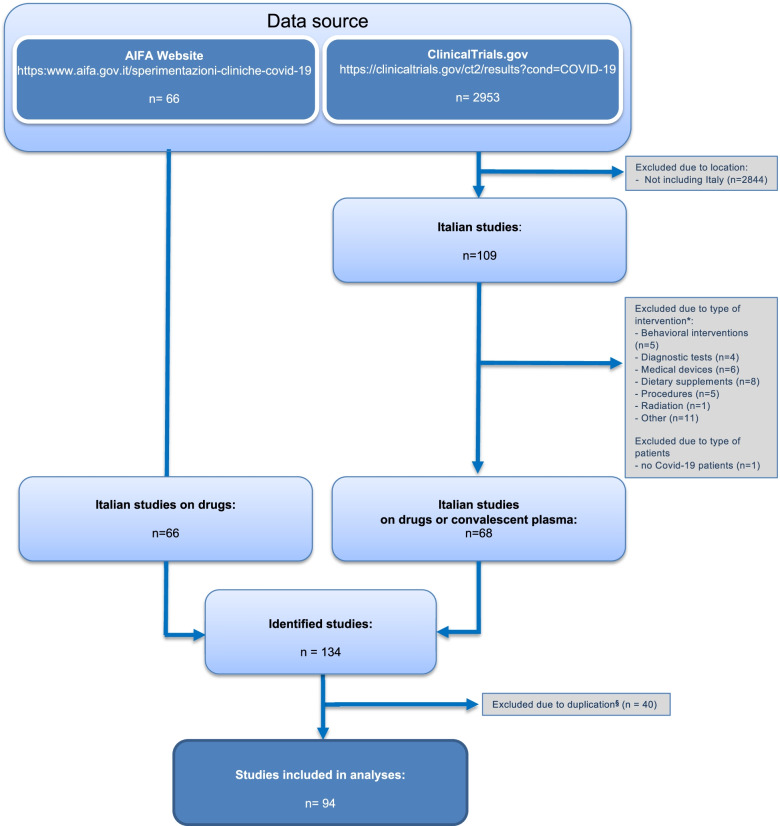
Identification of COVID-19 interventional clinical studies. Flow diagram of identification of COVID-19 interventional clinical studies on drugs or convalescent plasma planned to be carried out in Italy (update: 6 April 2021). Data source: AIFA website [15]; ClinicalTrials.gov [17]. *Assumption on experimental interventions: Escin (study identification number: NCT04475120) and lactoferrin (study identification number: NCT04322344) were included under the category dietary supplements; Nitric oxide (study identification numbers: NCT04290871 and NCT04290858) was included under the category “other”; Vitamin C (study identification number: NCT04323514) was included under the category drug taking into account dosage and route of administration. ^§^Clinical studies identified from ClinicalTrials.gov registry already identified from the AIFA website were considered duplicates and information reported on the AIFA website was considered for analyses

Figure 1 shows the detailed procedure used for the identification of the interventional clinical studies planned in Italy. On the 6th of April 2021, 66 pharmacological COVID-19 clinical trials were present in the AIFA website. At the same date, 109 clinical trials enrolling patients in Italy were registered at ClinicalTrials.gov. Among them, 41 studies were excluded accordingly to the pre-defined selection criteria. The NCT04475120 (Escin) and NCT04322344 (Lactoferrin) studies were included under the food supplements category. The NCT04290871 and NCT04290858 (Nitric oxide) studies were included under the category “Others,” while considering the dosage and intravenous route of administration, the NCT04323514 (Vitamin C) study was considered as drug. Of the remaining 68 studies, 40 were excluded being duplicated in the AIFA list.

Finally, we conducted our analysis on 94 clinical trials of which 66 (70%) were registered at the AIFA website and 28 (30%) were retrieved from ClinicalTrials.gov.

### Study characteristics

Table 1 shows the characteristics of the identified clinical trials both overall and separately for those listed in AIFA website and for those retrieved only on ClinicalTrials.gov registry. By considering all studies (*n*=94), 86 were therapeutic trials (91%) and 8 were preventive trials (9%). The majority of the studies were phase 2 (*n*=29, 34%), phase 2/3 (*n*=19, 22%), and phase 3 (*n*=32, 38%). The planned sample size was in the range of 101–1000 subjects in 53 (60%) trials, while 9 (10%) clinical studies envisaged the enrollment of more than 1000 patients (median sample size: 243 participants). Overall, 47,682 patients were planned to be recruited. With regard to the study design, 69 (74%) were parallel group trials, 70 (76%) were randomized, and 31 (33%) adopted masking procedures. The multicenter studies were 74 (80%) and 37 (40%) were multinational. Sponsor was pharmaceutical industry in 32 (34%) of the total studies.

**Table 1:** Study characteristics

Study characteristics	All studies *n*=94	Study source	
		AIFA website^a^ *n*=66	ClinicalTrials.gov^b^ *n*=28
*Primary purpose*, n (%)			
Treatment	86 (91%)	60 (91%)	26(93%)
Prevention	8 (9%)	6 (9%)	2 (7%)
*Phase*, n (%)			
1	3 (4%)	3 (5%)	-
1/2	1 (1%)	1 (2%)	
2	29 (34%)	20 (31%)	9 (43%)
2/3	19 (22%)	13 (20%)	6 (29%)
3	32 (38%)	26 (40%)	6 (29%)
4	1 (1%)	1 (2%)	-
Not reported	*1*	*1*	*-*
Not applicable	*8*	*1*	*7*
*Sample size*^*c*^, n (%)			
1-100	26 (30%)	14 (23%)	12 (46%)
101-1000	53 (60%)	39 (63%)	14 (54%)
>1000	9 (10%)	9 (14%)	-
Not reported	*6*	*4*	*2*
*Study design*			
Single group	20 (22%)	9 (14%)	11 (39%)
Parallel group	69 (74%)	55 (85%)	14 (50%)
Cross over	1 (1%)	-	1 (4%)
Sequential	3 (3%)	1 (1%)	2 (7%)
Not reported	*1*	*1*	*-*
*Randomization,* n (%)			
Yes	70 (76%)	55 (85%)	15 (56%)
No	22 (24%)	10 (15%)	12 (44%)
Not reported	*2*	*1*	*1*
*Masking*, n (%)			
Yes	31 (33%)	25 (38%)	6 (21%)
No	62 (67%)	40 (62%)	22 (79%)
Not reported	*1*	*1*	*-*
*Multicenter study,* n (%)			
Yes	74 (80%)	56 (88%)	18 (64%)
No	18 (20%)	8 (12%)	10 (36%)
Not reported	*2*	*2*	*-*
*International study,* n (%)			
Yes	37 (40%)	31 (48%)	6 (21%)
No	55 (60%)	33 (52%)	22 (79%)
Not reported	*2*	*2*	*-*
*Study sponsor,* n (%)			
Industry	32 (34%)	27 (41%)	5 (18%)
No industry	62 (66%)	39 (59%)	23 (82%)

Characteristics of COVID-19 interventional clinical trials planned to be carried out in Italy, overall and by data source (update: 6 April 2021)

^a^AIFA website [14]

^b^ClinicalTrials.gov [16]

^c^Sample size, median (Interquartile range): overall, 243 (355), Study source: AIFA website, 285 (334); Study source: ClinicalTrials.gov, 149 (273)

Among studies retrieved only on ClinicaTrials.gov registry (28), we noted that 12 (46%) planned to enroll less than 100 patients, 11 (39%) had a single group design, 15 (56%) studies were randomized, 6 were masked (21%), 18 (64%) were multicenter, 6 (21%) were international studies, and 5 (18%) were industry sponsored.

### Multiarm studies

Among 94 studies collected overall, 8 (9%) multiarm trials were identified (Table 2). All of these were multicentric trials and 6 out 8 (75%) had an Italian Sponsor. Two out of 8 (25%) multiarm trials were platform trials.

**Table 2:** Description of multiarm clinical trials

Study acronym	Experimental intervention	Sponsor country/Organization	Data source	Platform Trial
ACTIVE 4	Dalteparin, Enoxaparin, Heparin, Fondaparin, Tinzaparin	Italy	AIFA website	No
AMMURAVID	Remdesivir, Baricitinib, Canakinumab, Methylprednisolone, Sarilumab*, Siltuximab, Tocilizumab	Italy	AIFA website	No
ARCO	Darunavir/cobicistat, Favipiravir, Hydroxychloroquine, Lopinavir/Ritonavir	Italy	AIFA website	No
CONVINCE	Edoxaban, Colchicine	Switzerland, Italy	AIFA website	No
REMAP-CAP	ACE inhibitors, Acetylsalicylic acid, AZD7442, Angiotensin receptor blockers, Clopidogrel, Enoxaparin, Heparin, Interferon-beta-1a, Prasugrel, Sarilumab, Simvastatin, Ticagrelor, Tocilizumab, Vitamin C	Netherlands	AIFA website	Yes
SobiIMMUNO - 101	Emapalumab, Anakinra	Italy	AIFA website	No
SOLIDARITY	Chloroquine (or Hydroxychloroquine), Interferon beta-1a, Remdesivir, Lopinavir/Ritonavir	WHO	AIFA website	Yes
STAUNCH	Enoxaparin+Methylprednisolone, Heparin+Methylprednisolone	Italy	AIFA website	No

Details of multiarm COVID-19 interventional clinical trials planned to be carried out in Italy (update: 6 April 2021)

^*^For the study AMMURAVID, the experimental intervention Sarilumab was retrieved from the protocol v3, 17 April 2020. AIFA website [14]

### Experimental interventions and therapeutic classes

Table 3 shows the list of experimental interventions by considering all studies. Data source, identification number, title, and acronym have been listed for each trial investigating every experimental intervention. In detail, 63 different experimental interventions have been identified: 43 of them (68%) were investigated in single studies, 10 (16%) were present in 2 studies, and 10 (16%) were explored in at least 3 studies (Table 3). The most investigated treatment was convalescent plasma that was explored in 12 clinical trials, whereas 6 clinical trials planned to use hydroxychloroquine and other 6 were based on enoxaparin therapy. Noteworthy, among experimental interventions identified in Clinical Trials.gov registry not found in the AIFA database, in addition to the expected convalescent plasma and Vitamin C, some other drugs have been identified (Table 3).

**Table 3:** Experimental interventions: study details

Experimental intervention	Data source^§^	Study ID^#^	Study Title	Study acronym
ABX464	AIFA website	2020-001673-75	A phase 2/3, randomized, double blind, placebo-controlled study to evaluate the efficacy and the safety of ABX464 in treating inflammation and preventing COVID-19 associated acute respiratory failure in patients aged ≥ 65 and patients aged ≥18 with at least one additional risk factor who are infected with SARS-CoV-2. (the MiR-AGE study).	MiR-AGE
Acalabrutinib	AIFA website	2020-001644-25	A Phase 2, Open Label, Randomized Study of the Efficacy and Safety of Acalabrutinib with Best Supportive Care Versus Best Supportive Care in Subjects Hospitalized with COVID-19	ACE-ID-201
ACE inhibitors	AIFA website	2015-002340-14	Randomized, Embedded, Multifactorial Adaptive Platform trial for Community-Acquired Pneumonia (REMAP-CAP)	REMAP-CAP
Acetylsalicyclic acid	AIFA website	2015-002340-14	Randomized, Embedded, Multifactorial Adaptive Platform trial for Community-Acquired Pneumonia (REMAP-CAP)	REMAP-CAP
	ClinicalTrials.gov	NCT04808895	Acetylsalicylic Acid in the Prevention of Severe SARS-CoV2 Pneumonia in Hospitalised Patients With COVID-19	Asperum
Alteplase	ClinicalTrials.gov	NCT04640194	A Study to Test Whether Different Doses of Alteplase Help People With Severe Breathing Problems Because of COVID-19	TRISTARDS
Anakinra	AIFA website	2020-001167-93	A phase 2/3, randomized, open-label, parallel group, 3-arm, multicenter study investigating the efficacy and safety of intravenous administrations of emapalumab, an anti-interferon gamma (anti-IFNγ) monoclonal antibody, and anakinra, an interleukin-1(IL-1) receptor antagonist, versus standard of care, in reducing hyper-inflammation and respiratory distress in patients with SARSCoV-2 infection (Sobi.IMMUNO-101)	Sobi.IMMUNO-101
	AIFA website	2020-005828-11	Supar-guided anakinra treatment for validation of the risk and early management of severe respiratory failure by covid-19: the save-more double-blind, randomized, phase iii confirmatory trial	SAVE-MORE
	AIFA website	2015-002340-14	Randomized, Embedded, Multifactorial Adaptive Platform trial for Community-Acquired Pneumonia (REMAP-CAP)	REMAP-CAP
Angiotensin receptor blockers	AIFA website	2015-002340-14	Randomized, Embedded, Multifactorial Adaptive Platform trial for Community-Acquired Pneumonia (REMAP-CAP)	REMAP-CAP
AZD7442	AIFA website	2020-005315-44	A Phase III Randomized, Double-blind, Placebo-controlled, Multicenter Study to Determine the Safety and Efficacy of AZD7442 for the Treatment of COVID-19 in Non-hospitalized Adults	TACKLE Study
Baricitinib	AIFA website	2020-001955-42	BARICIVID-19 STUDY: MultiCentre, randomised, Phase IIa clinical trial evaluating efficacy and tolerability of Baricitinib as add-on treatment of in-patients with COVID-19 compared to standard therapy	BARCIVID
	AIFA website	2020-001854-23	Cumulative adaptive, multiarm, multistage and multicentre randomized clinical trial with immunotherapy for Moderate COVID-19	AMMURAVID
	AIFA website	2020-001517-21	A Randomized, Double-Blind, Placebo-Controlled, Parallel-Group Phase 3 Study of Baricitinib in Patients with COVID-19 Infection	COV-BARRIER
	AIFA website	2020-001185-11	A proof-of concept study of the use of Janus Kinase 1 and 2 Inhibitor, Baricitinib, in the treatment of COVID-19-related pneumonia	BREATH trial
	ClinicalTrials.gov	NCT04358614	Baricitinib Therapy in COVID-19	HPrato-4
Bevacizumab	Clincaltrials.gov	NCT04275414	Bevacizumab in Severe or Critical Patients With COVID-19 Pneumonia	BEST-CP
Canakinumab	AIFA website	2020-001370-30	Phase 3 multicenter, randomized, double-blind, placebocontrolled study to assess the efficacy and safety of canakinumab on cytokine release syndrome in patients with COVID-19-induced pneumonia (CAN-COVID)	CAN-COVID
	AIFA website	2020-001854-23	Cumulative adaptive, multiarm, multistage and multicentre randomized clinical trial with immunotherapy for Moderate COVID-19	AMMURAVID
Chloroquine	AIFA website	2020-001366-11	An international randomised trial of additional treatments for COVID-19 in hospitalised patients who are all receiving the local standard of care	SOLIDARITY
Cholecalciferol	AIFA website	2020-002119-23	COVitaminD Trial: prevenzione di complicanze da COVID-19 in pazienti oncologici in trattamento attivo	COVitaminD
Clopidogrel	AIFA website	2015-002340-14	Randomized, Embedded, Multifactorial Adaptive Platform trial for Community-Acquired Pneumonia (REMAP-CAP)	REMAP-CAP
Colchicine	AIFA website	2020-001475-33	Treatment with COLchicine of patients affected by COLVID-19 : a Pilot Study	COLVID-19
	AIFA website	2020-001258-23	Colchicine To Counteract Inflammatory Response In Covid-19 Pneumonia	ColCOVID
	AIFA website	2020-001806-42	ColcHicine in patients with COVID-19: a home CarE study	CHOICE-19
	AIFA website	2020-002234-32	Efficacy and Safety of Edoxaban and or Colchicine for patients with SARS-CoV-2 infection managed in the out of hospital setting (COVID 19)	CONVINCE
Convalescent plasma	ClinicalTrials.gov	NCT04385043	Hyperimmune Plasma in Patients With COVID-19 Severe Infection	COV2-CP
	ClinicalTrials.gov	NCT04428021	Standard or Convalescent Plasma in Patients With Recent Onset of COVID-19 Respiratory Failure	PLACO-COVID
	ClinicalTrials.gov	NCT04321421	Hyperimmune Plasma for Critical Patients With COVID-19	COV19-PLASMA
	ClinicalTrials.gov	NCT04346589	Convalescent Antibodies Infusion in Critically Ill COVID 19 Patients	
	ClinicalTrials.gov	NCT04393727	Transfusion of Convalescent Plasma for the Early Treatment of Patients With COVID-19	TSUNAMI
	ClinicalTrials.gov	NCT04418531	Convalescent Antibodies Infusion in COVID 19 Patients	
	ClinicalTrials.gov	NCT04374526	Early transfusIon of Convalescent Plasma in Elderly COVID-19 Patients. to Prevent Disease Progression.	LIFESAVER
	ClinicalTrials.gov	NCT04569188	Convalescent Plasma in COVID-19 Elderly Patients	RESCUE
	ClinicalTrials.gov	NCT04614012	Hyperimmune Plasma for Patients With COVID-19	IMMUNO-COVID19
	ClinicalTrials.gov	NCT04622826	plasmApuane CoV-2 : Efficacy and Safety of Immune Covid-19 Plasma in Covid-19 Pneumonia in Non ITU Patients	
	ClinicalTrials.gov	NCT04721236	Early Use of Hyperimmune Plasma in COVID-19	COV-II-PLA
	ClinicalTrials.gov	NCT04716556	TranSfUsion of coNvalescent plAsma for the Early Treatment of pneuMonIa in COVID-19 Patients	
COVID-eVax	AIFA website	2020-003734-20	A Phase I/Ii Study To Assess The Safety And Immunogenicity Of Covid-Evax, A Candidate Plasmid Dna Vaccine For Covid-19, In Healthy Adult Volunteers	COVID-eVax
CPI-006	ClinicalTrials.gov	NCT04734873	CPI-006 Plus Standard of Care Versus Placebo Plus Standard of Care in Mild to Moderately Symptomatic Hospitalized Covid-19 Patients	
CT-P59	AIFA website	2020-003401-60	A Phase 2/3, Randomized, Parallel-Group, Placebo-Controlled, Double-Blind Study to Evaluate the Efficacy and Safety of CT-P59 in Combination with Standard of Care in Hospitalized Patients with SARS-CoV-2 Infection	CT-P59 ospedalizzati
	AIFA website	2020-003369-20	CT-P59A Phase 2/3, Randomized, Parallel-group, Placebo-controlled, Double-Blind Study to Evaluate the Efficacy and Safety of CT-P59 in Combination with Standard of Care in Outpatients with Severe Acute Respiratory Syndrome Coronavirus (SARS-CoV-2) Infection. - CELLTRION	CT-P59 non ospedalizzati
Cyclosporin-A	AIFA website	2020-003505-58	A proof-of-concept study of the use of Inhaled liposomal -Cyclosporin-A in the treatment of moderate COVID-19-related pneumonia: a two-step phase II clinical trial	INCIPIT
Dalteparin	AIFA website	2020-004285-19	A Multicenter Adaptive Randomized Controlled Platform Trial of the Safety and Efficacy of Antithrombotic Strategies in Hospitalized Adults with COVID-19	ACTIVE4
Darunavir/cobicistat	AIFA website	2020-001528-32	Adaptive Randomized trial for therapy of COrona virus disease 2019 at home with oral antivirals (ARCO-Home study)	ARCO
DAS181	ClinicalTrials.gov	NCT04354389	DAS181 for STOP COVID-19	
Defibrotide	AIFA website	2020-001513-20	Use of Defibrotide to reduce progression of acute respiratory failure rate in patients with COVID-19 pneumonia	DEF-IVID19
Edoxaban	AIFA website	2020-002234-32	Efficacy and Safety of Edoxaban and or Colchicine for patients with SARS-CoV-2 infection managed in the out of hospital setting (COVID 19)	CONVINCE
Emapalumab	AIFA website	2020-001167-93	A phase 2/3, randomized, open-label, parallel group, 3-arm, multicenter study investigating the efficacy and safety of intravenous administrations of emapalumab, an anti-interferon gamma (anti-IFNγ) monoclonal antibody, and anakinra, an interleukin-1(IL-1) receptor antagonist, versus standard of care, in reducing hyper-inflammation and respiratory distress in patients with SARSCoV-2 infection (Sobi.IMMUNO-101)	Sobi.IMMUNO-101
Enoxaparin	AIFA website	2020-001708-41	Enoxaparina for thromboprophylaxis in hospitalized COVID-19 patients: comparison of 40mg o.d. versus 40mg b.i.d. A randomized Clinical Trial	X-COVID
	AIFA website	2020-001972-13	Randomised controlled trial comparing efficacy and safety of high versus low Low- Molecular Weight Heparin dosages in hospitalised patients with severe COVID-19 pneumonia and coagulopathy not requiring invasive mechanical ventilation (COVID-19 HD)	COVID-19 HD
	AIFA website	2020-001308-40	Intermediate dose enoxaparin in hospitalized patients with moderate-severe COVID19: a pilot phase II single-arm study, INHIXACOVID19	INHIXACOVID
	AIFA website	2020-002214-40	EMOS-COVID - Enoxaparina	EMOS-COVID
	AIFA website	2020-004285-19	A Multicenter Adaptive Randomized Controlled Platform Trial of the Safety and Efficacy of Antithrombotic Strategies in Hospitalized Adults with COVID-19	ACTIVE4
	AIFA website	2015-002340-14	Randomized, Embedded, Multifactorial Adaptive Platform trial for Community-Acquired Pneumonia (REMAP-CAP)	REMAP-CAP
Enoxaparin+Methylprednisolone	AIFA website	2020-001921-30	Steroids and unfractionated heparin in critically ill patients with pneumonia from COVID-19 infection. A multicenter, interventional, randomized, three arms study design.	STAUNCH
Favipiravir	AIFA website	2020-001528-32	Adaptive Randomized trial for therapy of COrona virus disease 2019 at home with oral antivirals (ARCO-Home study)	ARCO
	AIFA website	2020-001115-25	A Multi-center, Randomized, Double-blind, Placebo-controlled, Phase III Clinical Study Evaluating the Efficacy and Safety of Favipiravir in the Treatment of Adult Inpatients with COVID-19-General Type (HS216C17	HS216C17
Fondaparin	AIFA website	2020-004285-19	A Multicenter Adaptive Randomized Controlled Platform Trial of the Safety and Efficacy of Antithrombotic Strategies in Hospitalized Adults with COVID-19	ACTIVE4
GRAd-COV2	AIFA website	2020-002835-31	A Phase 1, Dose-Escalation Study to assess the Safety and Immunogenicity of a COVID-19 Vaccine GRAd-COV2 in Healthy Adults and Elderly Subjects	RT-CoV-2
	AIFA website	2020-005915-39	A Phase II/III, Randomized, Stratified, Observer-Blind, Placebo-Controlled Study to Evaluate the Efficacy, Safety, and Immunogenicity of GRAd-COV2 Vaccine in Adults Aged 18 Years and Older. COVITAR	COVITAR
Heparin	AIFA website	2020-004285-19	A Multicenter Adaptive Randomized Controlled Platform Trial of the Safety and Efficacy of Antithrombotic Strategies in Hospitalized Adults with COVID-19	ACTIVE4
	AIFA website	2015-002340-14	Randomized, Embedded, Multifactorial Adaptive Platform trial for Community-Acquired Pneumonia (REMAP-CAP)	REMAP-CAP
Heparin+Methylprednisolone	AIFA website	2020-001921-30	Steroids and unfractionated heparin in critically ill patients with pneumonia from COVID-19 infection. A multicenter, interventional, randomized, three arms study design.	STAUNCH
Hydroxycloroquine	AIFA website	2020-001441-39	Chloroquine/ hydroxychloroquine prevention of coronavirus disease (COVID-19) in the healthcare setting; a randomised, placebo-controlled prophylaxis study (COPCOV)	COP-COV
	AIFA website	2020-001987-28	PRECOV Idrossiclorochina negli operatori sanitari	PRECOV
	AIFA website	2020-001501-24	PROTECT: A randomized study with Hydroxychloroquine versus observational support for prevention or early phase treatment of Coronavirus disease (COVID-19)	PROTECT
	AIFA website	2020-001558-23	Hydroxychloroquine sulfate early administration in symptomatic out of hospital COVID-19 positive patients (Hydro-Stop-COVID19 Trial)	Hydro-Stop
	AIFA website	2020-001528-32	Adaptive Randomized trial for therapy of COrona virus disease 2019 at home with oral antivirals (ARCO-Home study)	ARCO
	AIFA website	2020-001366-11	An international randomised trial of additional treatments for COVID-19 in hospitalised patients who are all receiving the local standard of care	SOLIDARITY
Hydroxycloroquine+Azitromycin	AIFA website	2020-001802-50	AZI-RCT-COVID-19 - Studio sull'utilizzo di idrossiclorochina+azitromicina	AZI-RCT-COVID-19
hzVSF-v13	AIFA website	2020-003614-13	Efficacy and safety of intravenously administered hzVSF-v13 in patients with COVID-19 pneumonia: a phase II, proof of concept, multicentre, randomized, parallel-group, double-blind, placebo-controlled study	hzVSF_v13-0006
Interferon beta-1a	AIFA website	2020-001366-11	An international randomised trial of additional treatments for COVID-19 in hospitalised patients who are all receiving the local standard of care	SOLIDARITY
	AIFA website	2020-002458-25	Randomized, controlled, open label, phase 2 clinical trial of Interferon-β-1a (IFNβ-1a) in COVID-19 patients.	INTERCOP
	AIFA website	2020-003872-42	Antiviral and Immunomodulatory Interferon-Beta in high-risk COVID-19 patients	ANTIICIPATE
	AIFA website	2015-002340-14	Randomized, Embedded, Multifactorial Adaptive Platform trial for Community-Acquired Pneumonia (REMAP-CAP)	REMAP-CAP
Ivermectin	AIFA website	2020-002283-32	Randomized, Double-blind, Multi entre Phase II, Proof of Concept, Dose Finding Clinical Trial on Ivermectin for the early Treatment of COVID-19	COVER
Lopinavir/Ritonavir	AIFA website	2020-001528-32	Adaptive Randomized trial for therapy of COrona virus disease 2019 at home with oral antivirals (ARCO-Home study)	ARCO
	AIFA website	2020-001366-11	An international randomised trial of additional treatments for COVID-19 in hospitalised patients who are all receiving the local standard of care	SOLIDARITY
MAD0004J08	AIFA website	2020-005469-15	COVID-19: A Phase I dose-escalation study to evaluate the safty and pharmacokinetics of anti-SARS-CoV-2 monoclonal antibody MAD0004j08 in healthy adultse	MAD0004J08
Mavrilimumab	AIFA website	2020-001795-15	A randomized, double blind, placebo-COntrolled trial of MavrilimumaB for Acute respiratory failure due To COVID-19 pneumonia with hyper-inflammation: the COMBAT-19 trial	COMBAT-19
Methyilprednisolone	AIFA website	2020-001854-23	Cumulative adaptive, multiarm, multistage and multicentre randomized clinical trial with immunotherapy for Moderate COVID-19	AMMURAVID
	AIFA website	2020-004323-16	Uno studio randomizzato multicentrico in doppio cieco per valutare l'efficacia della somministrazione di Metilprednisolone ad alte dosi in aggiunta al trattamento standard in pazienti affetti da polmonite da SARS-CoV2 - Codice: RCT-MP-COVID-19	RCT-MP-COVID-19
	ClinicalTrials.gov	NCT04636671	Methylprednisolone vs. Dexamethasone in COVID-19 Pneumonia (MEDEAS RCT)	MEDAS
MK-4482	AIFA website	2020-003367-26	A Phase 2/3, Randomized, Placebo-Controlled, Double-Blind Clinical Study to Evaluate the Efficacy, Safety, and Pharmacokinetics of MK-4482 in Hospitalized Adults with COVID-19	MK-4482 ospedalizzati
	AIFA website	2020-003368-24	“A Phase 2/3, Randomized, Placebo-Controlled, Double-Blind Clinical Study to Evaluate the Efficacy, Safety, and Pharmacokinetics of MK-4482 in Non-Hospitalized Participants ≥18 Years of Age with COVID-19”	MK-4482 non ospedalizzati
Nafamostat Mesylate	ClinicalTrials.gov	NCT04352400	Efficacy of Nafamostat in Covid-19 Patients (RACONA Study)	RACONA
Opaganib	AIFA website	2020-002677-95	Opaganib, a Sphingosine Kinase-2 (SK2) Inhibitor in COVID-19 Pneumonia: a Randomized, Double-blind, Placebo-Controlled Phase 2/3 Study, in Adult Subjects Hospitalized with Severe SARS-CoV-2 Positive Pneumonia	ABC-201
Oxytocin	ClinicalTrials.gov	NCT04386447	Phase II RCT to Assess Efficacy of Intravenous Administration of Oxytocin in Patients Affected by COVID-19	OsCOVID19
Pamrevlumab	AIFA website	2020-001472-14	An Open-Label, Randomized, Parallel-Arm Study Investigating The Efficacy And Safety Of Intravenous Administration Of Pamrevlumab Versus Standard Of Care In Patients With Covid-19	FibroCov
Plitidepsin	ClinicalTrials.gov	NCT04784559	Trial to Determine the Efficacy/Safety of Plitidepsin vs Control in Patients With Moderate COVID-19 Infection	Neptuno
Prasugrel	AIFA website	2015-002340-14	Randomized, Embedded, Multifactorial Adaptive Platform trial for Community-Acquired Pneumonia (REMAP-CAP)	REMAP-CAP
	ClinicalTrials.gov	NCT04445623	Prasugrel in Severe COVID-19 Pneumonia	PARTISAN
Polyvalent immunoglobulins	AIFA website	2020-002058-26	High dose intravenous polyvalent immunoglobulin (IVIG) in patients with early inflammatory COVID-19.	IVIG/H/Covid-19
Raloxifene	AIFA website	2020-003936-25	Multicenter, adaptive, randomized, placebo-controlled, double blind, parallel-group Phase 2/3 trial, to study efficacy and safety of two doses of raloxifene in adult paucisymptomatic COVID- 19 patients.	RLX0120
Ravulizumab	AIFA website	2020-001497-30	A Phase 3 Open-label, Randomized, Controlled Study to Evaluate the Efficacy and Safety of Intravenously Administered Ravulizumab Compared with Best Supportive Care in Patients with COVID-19 Severe Pneumonia, Acute Lung Injury, or Acute Respiratory Distress Syndrome	ALXN1210-COV-305
Remdesivir	AIFA website	2020-000842-32	A Phase 3 Randomized Study to Evaluate the Safety and Antiviral Activity of Remdesivir (GS-5734™) in Participants with Moderate COVID-19 Compared to Standard of Care Treatment. (GS-US-540-5774 Study)	GS-US-540-5774
	AIFA website	2020-000841-15	A Phase 3 Randomized Study to Evaluate the Safety and Antiviral Activity of Remdesivir (GS-5734™) in Participants with Severe COVID-19. (GS-US-540-5773 Study)	GS-US-540-5773
	AIFA website	2020-001366-11	An international randomised trial of additional treatments for COVID-19 in hospitalised patients who are all receiving the local standard of care	SOLIDARITY
	AIFA website	2020-001803-17	A Phase 2/3 Single-Arm, Open-Label Study to Evaluate the Safety, Tolerability, Pharmacokinetics, and Efficacy of Remdesivir (GS-5734™) in Participants from Birth to < 18 Years of Age with COVID-19 (GS-US-540-5823)	GS-US-540-5823
Reparixin	AIFA website	2020-001645-40	Adaptive phase 2/3, randomized, controlled multicenter study on the efficacy and safety of Reparixin in the treatment of hospitalized patients with COVID-19 pneumonia (REPAVID-19)	REPAVID-19
	AIFA website	2020-005919-51	Studio di fase 3, multicentrico, randomizzato, controllato con placebo, sull'efficacia e la sicurezza di Reparixin nel trattamento di pazienti ospedalizzati con polmonite grave da COVID-19	REPAVID-19 Phase 3
Ruxolitinib	AIFA website	2020-001662-11	Adaptive phase 2/3, randomized, controlled multicenter study on the efficacy and safety of Reparixin in the treatment of hospitalized patients with COVID-19 pneumonia (REPAVID-19)	RUXCOVID
Sarilumab	AIFA website	2020-001390-76	ESCAPE Studio di fase 2 sull'utilizzo di sarilumab	ESCAPE
	AIFA website	2020-001162-12	An adaptive phase 2/3, randomized, double-blind, placebocontrolled study assessing efficacy and safety of sarilumab for hospitalized patients with COVID-19 (Sarilumab COVID-19).	Sarilumab COVID-19
	AIFA website	2020-001854-23	Cumulative adaptive, multiarm, multistage and multicentre randomized clinical trial with immunotherapy for Moderate COVID-19	AMMURAVID^*^
	AIFA website	2020-001745-40	Pilot study on the use of sarilumab in patients with covid-19 infection (COVID-SARI)	COVID-SARI
	AIFA website	2015-002340-14	Randomized, Embedded, Multifactorial Adaptive Platform trial for Community-Acquired Pneumonia (REMAP-CAP)	REMAP-CAP
Selinexor	AIFA website	2020-001411-25	A Phase 2 Randomized Single-Blind Study to Evaluate the Activity and Safety of Low Dose Oral Selinexor (KPT-330) in Patients with Severe COVID-19 Infection (XPORT-CoV-1001)	XPORT-CoV-1001
Siltuximab	AIFA website	2020-001854-23	Cumulative adaptive, multiarm, multistage and multicentre randomized clinical trial with immunotherapy for Moderate COVID-19	AMMURAVID
Simvastatin	AIFA website	2015-002340-14	Randomized, Embedded, Multifactorial Adaptive Platform trial for Community-Acquired Pneumonia (REMAP-CAP)	REMAP-CAP
Sitagliptin	ClinicalTrials.gov	NCT04365517	The Effect of Sitagliptin Treatment in COVID-19 Positive Diabetic Patients	SIDIACO
Ticagrelor	AIFA website	2015-002340-14	Randomized, Embedded, Multifactorial Adaptive Platform trial for Community-Acquired Pneumonia (REMAP-CAP)	REMAP-CAP
Tinzaparin	AIFA website	2020-004285-19	A Multicenter Adaptive Randomized Controlled Platform Trial of the Safety and Efficacy of Antithrombotic Strategies in Hospitalized Adults with COVID-19	ACTIVE4
Tirofiban	ClinicalTrials.gov	NCT04368377	Enhanced Platelet Inhibition in Critically Ill Patients With COVID-19	PIC-19
Tocilizumab	AIFA website	2020-001110-38	Multicenter study on the efficacy and tolerability of tocilizumab in the treatment of patients with COVID-19 pneumonia (TOCIVID-19)	TOCIVID-19
	AIFA website	2020-001386-37	RCT-TCZ-COVID-19 somministrazione precoce del Tocilizumab	RCT-TCZ-COVID-19
	AIFA website	2020-001154-22	A randomized, double-blind, placebocontrolled, multicenter study to evaluate the safety and efficacy of tocilizumab in patients with severe covid-19 pneumonia (Tocilizumab 2020-001154-22)	Tocilizumab 2020-001154-22
	AIFA website	2020-001854-23	AMMURAVID Studio di fase 3 multiarm della SIMIT	AMMURAVID
	AIFA website	2020-005291-35	A multicenter randomized trial to evaluate the efficacy of tocilizumab in patients with severe Coronavirus Disease 2019 (Covid-19) pneumonia failing glucocorticoids (Anticipant Study)	ANTICIPANT Study
	AIFA website	2015-002340-14	Randomized, Embedded, Multifactorial Adaptive Platform trial for Community-Acquired Pneumonia (REMAP-CAP)	REMAP-CAP
	ClinicalTrials.gov	NCT04315480	Tocilizumab for SARS-CoV2 (COVID-19) Severe Pneumonitis	
Tofacitinib	AIFA website	2020-002035-30	TOFAcitinib plus Hydroxycloroquine vs Hydroxycloroquine in patients with early onset SARS-CoV2 (COVID-19) interstitial pneumonia: a multicenter randomized controlled open label trial	TOFACOV-2
	ClinicalTrials.gov	NCT04332042	TOFAcitinib in SARS-CoV2 Pneumonia	TOFACOV
Vitamin C	AIFA website	2015-002340-14	Randomized, Embedded, Multifactorial Adaptive Platform trial for Community-Acquired Pneumonia (REMAP-CAP)	REMAP-CAP
	ClinicalTrials.gov	NCT04323514	Use of Ascorbic Acid in Patients With COVID 19	

Experimental interventions investigated in COVID-19 interventional clinical trials planned to be carried out in Italy (update: 6 April 2021)

^§^AIFA website (14); ClinicalTrials.gov [16]

^#^Study ID is EudraCT Number for study from AIFA website and NCT Number for study from ClinicalTrials.gov website

^*^For the study AMMURAVID, the experimental intervention Sarilumab was retrieved from the protocol v3, 17 April 2020

Therapeutic classes and subclasses of experimental interventions are described in Table S1. The most frequent classes are immune-suppressors/immune-modulators (*n*=36), anti-thrombotic/anti-coagulants (*n*=22), plasma-derived (*n*=12), anti-viral (*n*=13), anti-malaria (*n*=7), and antibodies against SARS-CoV-2 (*n*=5). We have also identified 2 candidate vaccines: GRAd-COV2 with two trials (a phase I trial—EudractCT Number 2020-002835-31, and a phase II/III trial—EudractCT Number 2020-005915-39) and COVID-eVax with one trial (a phase I/II trial—EudractCT Number 2020-003734-20).

Notably, the identified clinical trials are focused on the use of molecules that belong to very different therapeutic classes (anti-osteoporotic, anti-diabetic, anti-gout, anti-hypertensive, anti-tumoral, hormones, statins), thus suggesting a drug repositioning approach.

## Discussion

In response to the global coronavirus infection (COVID-19) emergency, a huge number of clinical trials exploring a variety of interventions have been proposed by the scientific community worldwide in order to search for efficacious therapeutic approaches. The main purpose of the present work was to analyze the landscape of COVID-19 clinical research in Italy, by mapping and describing the characteristics of planned clinical trials investigating the role of drugs and convalescent plasma for treatment or prevention of COVID-19 disease. In Italy, the AIFA was entrusted with the task of managing the submission and authorization process of all clinical trials on medicines for the treatment and prevention of COVID-19. Therefore, similarly to other National Competent Authorities in Europe, AIFA set up simplified, accelerated procedures (“Cura Italia” Decree, 18 March 2020 [12]). A list of all authorized studies, including full protocols, is regularly updated and publicly accessible in the AIFA, thereby providing the opportunity for mapping and tracking COVID-19 clinical trials. To our knowledge, not all Competent Authorities in Europe have provided open access to the same amount of information.

Unfortunately, information on pharmacological clinical trials that are still under evaluation or have been denied authorization is not made available on AIFA website. Moreover, studies on non-pharmacological interventions, even if submitted at the single National Ethics Committee, do not undergo AIFA evaluation. We therefore extended our search to the ClinicalTrials.gov registry, which is widely used by investigators and sponsors worldwide and reports also some information on the study protocols. Data from the two sources were properly integrated (see the “Methods” section) to avoid duplication.

At 6 April 2021, at about 1 year since the WHO declaration of COVID-19 pandemic, we identified a considerable number of clinical trials on drugs or plasma from convalescent patients planned to be carried out in Italy, thus being aligned with international activities [1–6].

Overall, our survey showed that some worthwhile clinical characteristics were widely implemented (parallel groups, randomization), whereas other were frequently missed (blinding), likely suggesting difficulties concerning organization and conduction (such as packaging and labeling experimental products or preparing placebo) [7]. The majority of clinical trials reported considerable sample sizes (>100). Moreover, most of the studies were multicenter, indicating collaboration efforts among clinical sites, even at the international level, and active participation of Italian investigators in multinational networks. In fact, over the time, a progressive shift from small single site studies to multicenter, national, and international collaborative clinical trials (including multiarm studies) was observed (data not shown). Indeed, the networking among clinical researchers proposing similar experimental interventions was strongly supported also by AIFA [20]. However, evaluating whether this evolution has impacted on the quality and efficiency in the conduct of the studies is beyond the scope of our work.

Of note, several clinical trials were retrieved only from ClinicalTrials.gov registry. They were mainly single group design, unblinded, non-randomized, and small sample size studies, suggesting possible methodological issues for some of them. Moreover, multicenter and international clinical trials were less represented. By looking at experimental intervention, about half of them were focused on convalescent plasma that in Italy is not classified as “drug” and is under the technical and scientific control of the National Blood Center. Although the study on ClinicalTrials.gov NCT04323514 reported Vitamin C treatment as dietary supplement, we include the study in the survey as considering of the high dosage and the route of administration (intravenous) which determine its intended use as a drug and not as a supplement. Some explanations for the others pharmacological clinical trials found in the ClinicalTrials.gov website and not in the AIFA list might be hypothesized: they may represent studies still under evaluation or just planned, or even studies that have been denied authorization but were not removed from the website.

We found that some experimental interventions were investigated contemporaneously in different studies. This might be useful to build up a body of evidence to evaluate the efficacy of a given intervention, but might also lead to a fragmentation of the research and sub-optimal use of resources. This should be avoided in a pandemic scenario where the identification of potential treatments is an unmet need requiring “quick” responses by means of coordinated efforts and rigorously conducted clinical studies [7]. The use of “convalescent plasma” to prevent COVID-19 disease progression [21] may be taken as an example. In Italy, to provide a rapid and scientifically founded answer on the role of this approach, a national, randomized multicenter trial was carried out under the sponsorship of ISS and AIFA (TSUNAMI study, NCT04716556). This allowed the enrolment of 487 hospitalized patients with COVID-19 pneumonia from 27 clinical sites in Italy in less than 5 months (15 July to 8 December 2020) [22].

The characteristics of therapeutic interventions deserve some considerations. At the beginning of the COVID-19 outbreak, in Italy as everywhere in the world, most clinical trials were based on repositioning of drugs already approved for other indications and prescribed through a systematic off-label use. Consistently, most studies were classified as phase 2, phase 2/3, or phase 3. The most frequently reported therapeutic classes included antimalarial agents (prescribed on the basis of previous experience in SARS-COV 1 and MERS-COV diseases), immunosuppressant and immune-modulating drugs, anticoagulants, immunoglobulin, and antivirals used for other infections, such as lopinavir/ritonavir and favipiravir.

Regarding the experimental intervention, a shift in the distribution of therapeutic classes was observed over time [23], due to the lack of efficacy of some repurposed drugs (such as chloroquine) and the scientific knowledge acquired during the pandemic.

In the absence of an effective therapy, several studies were based on dietary supplements. The distinction between dietary supplement and drug is often faded, since the classification of a product in one of the two categories depends on various factors such as the type of substance, the dosage and the route of administration and, above all, the purpose of use [24]. Regulatory aspects reflect this ambiguity and thus it is not surprising that some Italian clinical trials, detected in ClinicalTrials.gov and for which the sponsor indicates the active intervention as “dietary supplement,” have never been submitted to the AIFA evaluation.

As expected, clinical trials submitted to AIFA included also preventive interventions with 2 candidate COVID-19 vaccines. A phase I study with the GRAd-COV2 vaccine was approved on 29 July 2020 and reached phase 2/3 on 19 February 2021. An additional phase I/II study was authorized on 03 February 2021 for COVID-EVAX vaccine.

A final analysis regards the trial sponsorship, with non-industrial sponsors, such as academic or public hospitals, supporting majority of the studies. This heavy involvement of nonprofit institutions indicates the willingness of investigators to promptly face the emergency taking advantage of previous experiences (even if limited) and of the available knowledge.

Our work has some limitations. We used ClinicaTrials.gov as source of data to integrate the information found in the AIFA website. However, there are some issues with this registry which need to be considered because the information reported in ClinicaTrials.gov—differently from study protocols submitted to regulatory authority—may be partial, not undergoing assessment for authorization, and not always updated [25]. A further limit is that we were unable to capture potential changes in the studies due to protocol amendments. However, due to the limited time of observation, we may reasonably hypothesize that such changes occurred in a small number of trials with a minor impact on their major features. In addition, we do not know the total number of proposals submitted to AIFA, the number of rejections, and the number of studies on hold waiting for resolution of queries.

Our mapping of Italian clinical trials allowed to analyze the early response of the clinical research community to the national and worldwide COVID-19 emergency. The deep investigation highlighted a wide clinical research landscape. The analyses of study design characteristics have showed that high level quality clinical trials were planned although some weaknesses have been observed in line with international activities. Therefore, our study suggests that coordination and collaboration, as well as expert clinical methodologists, are needed in health emergency.

## Conclusions

To our knowledge, this is the first mapping of therapeutic clinical trials for COVID-19 disease performed in Italy since the beginning of the pandemic. This work was made possible due to the availability of study documents in the AIFA website which represents an important source of detailed information for sponsors, clinical researchers, and health care professionals and a stimulus to improve both clinical methodologies and planning of future research objectives.

In perspective, the following aspects can be analyzed: (i) description of the study endpoints and the populations included in the protocols, (ii) relationship between the methodological characteristics and the sponsorship, and (iii) monitoring of the trial completion and publication of results. This last point would be crucial in order to evaluate the fulfillment of the identified trials and, most importantly, the quality and efficacy of the clinical research strategy.

## Supplementary Information

**Additional file 1: Table S1.** Therapeutic classes and subclasses.

## Data Availability

The datasets used and/or analyzed during the current study are available from the authors on reasonable request.

## Abbreviations

AIFA: Italian Medicines Agency
ATC: Anatomical Therapeutical Chemical Classification System
COVID-19: Coronavirus disease 2019
CTS: Technical Scientific Committee
INMI: National Institute for Infectious Diseases
