# Supplementary material for: Drugs and convalescent plasma therapy for COVID-19: a survey of the interventional clinical studies in Italy after 1 year of pandemic
Source: Trials. 2022 Jun 22;23:527. doi: 10.1186/s13063-022-06474-8 (PMC9214678; doi:10.1186/s13063-022-06474-8)
Supplement: Supplementary file 1 — Additional file 1: Table S1. Therapeutic classes and subclasses. [file 13063_2022_6474_MOESM1_ESM.docx]

**Table S1 Therapeutic classes and subclasses**

| **Therapeutic class** | **Therapeutic subclass** | **Drugs/ Convalescent plasma** | **Data source^§^** | **Study ID^#^** | **Study acronym** |
| --- | --- | --- | --- | --- | --- |
| Antibiotics | Macrolide | Azithromicyn | AIFA website | 2020-001802-50 | AZI-RCT-COVID-19 |
| Anti SARS-CoV-2 antibodies |  | CT-P59 | AIFA website | 2020-003401-60 | CT-P59 ospedalizzati |
|  |  |  |  | 2020-003369-20 | CT-P59 non ospedalizzati |
|  |  | AZD7442 | AIFA website | 2020-005315-44 | TACKLE Study |
|  |  | hzVSF-v13 | AIFA website | 2020-003614-13 | hzVSF_v13-0006 |
|  |  | MAD0004J08 | AIFA website | 2020-005469-15 | MAD0004J08 |
| Antidiabetics |  | Sitagliptin | ClinicalTrials.gov | NCT04365517 | SIDIACO |
| Antigout |  | Colchicine | AIFA website | 2020-001475-33 | COLVID-19 |
|  |  |  |  | 2020-001258-23 | Col-COVID |
|  |  |  |  | 2020-001806-42 | CHOICE-19 |
|  |  |  |  | 2020-002234-32 | CONVICE |
| Antimalarials |  | Hydroxychloroquine | AIFA website | 2020-001441-39 | COP-COV |
|  |  |  |  | 2020-001987-28 | PRECOV |
|  |  |  |  | 2020-001501-24 | PROTECT |
|  |  |  |  | 2020-001558-23 | Hydro-Stop |
|  |  |  |  | 2020-001528-32 | ARCO |
|  |  |  |  | 2020-001366-11 | SOLIDARITY |
|  |  |  |  | 2020-001802-50 | AZI-RCT-COVID-19 |
|  |  | Chloroquine | AIFA website | 2020-001366-11 | SOLIDARITY |
| Antiosteoporotics |  | Cholecalciferol | AIFA website | 2020-002119-23 | COVitaminD |
|  |  | Raloxifen | AIFA website | 2020-003936-25 | RLX0120 |
| Antiparasitic |  | Ivermectin | AIFA website | 2020-002283-32 | COVER |
| Antihypertensives |  | ACE inhibitors | AIFA website | 2015-002340-14 | REMAP-CAP |
|  |  | Angiotensin receptor blockers | AIFA website | 2015-002340-14 | REMAP-CAP |
| Antithrombotics/anticoagulants |  | Heparin (unfractionated) | AIFA website | 2015-002340-14 | REMAP-CAP |
|  |  |  |  | 2020-001921-30 | STAUNCH |
|  |  |  |  | 2020-004285-19 | ACTIVE4 |
|  |  | Enoxaparin | AIFA website | 2020-001708-41 | X-COVID |
|  |  |  |  | 2020-001972-13 | COVID-19 HD |
|  |  |  |  | 2020-001308-40 | INHIXACOVID |
|  |  |  |  | 2020-002214-40 | EMOS-COVID |
|  |  |  |  | 2020-004285-19 | ACTIVE-4 |
|  |  |  |  | 2020-001921-30 | STAUNCH |
|  |  |  |  | 2015-002340-14 | REMAP-CAP |
|  |  | Defibrotide | AIFA website | 2020-001513-20 | DEF-IVID19 |
|  |  | Tirofiban | ClinicalTrials.gov | NCT04368377 | PIC-19 |
|  |  | Tinzaparin | AIFA website | 2020-004285-19 | ACTIVE4 |
|  |  | Dalteparin | AIFA website | 2020-004285-19 | ACTIVE4 |
|  |  | Fondaparin | AIFA website | 2020-004285-19 | ACTIVE4 |
|  |  | Prasugrel | AIFA website | 2015-002340-14 | REMAP-CAP |
|  |  |  | ClinicalTrials.gov | NCT04445623 | PARTISAN |
|  |  | Edoxaban | AIFA website | 2020-002234-32 | CONVINCE |
|  |  | Acetylsalicylic acid | AIFA website | 2015-002340-14 | REMAP-CAP |
|  |  |  | ClinicalTrials.gov | NCT04808895 | Asperum |
|  |  | Clopidogrel | AIFA website | 2015-002340-14 | REMAP-CAP |
|  |  | Ticagrelor | AIFA website | 2015-002340-14 | REMAP-CAP |
|  |  | Alteplase | ClinicalTrials.gov | NCT04640194 | TRISTARDS |
| Antitumor |  | Acalabrutinib | AIFA website | 2020-001644-25 | ACE-ID-201 |
|  |  | Bevacizumab | ClincalTrials.gov | NCT04275414 | BEST-CP |
|  |  | Selinexor | AIFA website | 2020-001411-25 | XPORT-CoV-1001 |
|  |  | Opaganib | AIFA website | 2020-002677-95 | ABC-110 |
|  |  | Plitidepsin | ClinicalTrials.gov | NCT04784559 | Neptuno |
| Antiviral |  | Favipiravir | AIFA website | 2020-001528-32 | ARCO |
|  |  |  |  | 2020-001115-25 | HS216C17 |
|  |  | Lopinavir | AIFA website | 2020-001528-32 | ARCO |
|  |  |  |  | 2020-001366-11 | SOLIDARITY |
|  |  | Ritonavir | AIFA website | 2020-001528-32 | ARCO |
|  |  |  |  | 2020-001366-11 | SOLIDARITY |
|  |  | Remdesivir | AIFA website | 2020-000842-32 | GS-US-540-5774 |
|  |  |  |  | 2020-000841-15 | GS-US-540-5773 |
|  |  |  |  | 2020-001366-11 | SOLIDARITY |
|  |  |  |  | 2020-001803-17 | GS-US-540-5823 |
|  |  | Darunavir | AIFA website | 2020-001528-32 | ARCO |
|  |  | MK4482 | AIFA website | 2020-003367-26 | MK-ospedalizzati |
|  |  |  |  | 2020-003368-24 | MK- non ospedalizzati |
| Immunoglobulins |  | Polyvalent immunoglobulins | AIFA website | 2020-002058-26 | IVIG/H/Covid-19 |
| Immunosoppressants/ Immunomodulators | Selective immunosuppressants | Baricitinib | AIFA website | 2020-001955-42 | BARCIVID |
|  |  |  |  | 2020-001854-23 | AMMURAVID |
|  |  |  |  | 2020-001517-21 | COV-BARRIER |
|  |  |  |  | 2020-001185-11 | BREATH trial |
|  |  |  | ClinicalTrials.gov | NCT04358614 | HPrato-4 |
|  |  | Tofacitinib | AIFA website | 2020-002035-30 | TOFACOV-2 |
|  |  |  | ClinicalTrials.gov | NCT04332042 | TOFACOV |
|  |  | Cyclosporin-A | AIFA website | 2020-003505-58 | INCIPIT |
|  |  | Ruxolitinib | AIFA website | 2020-001662-11 | RUXCOVID |
|  |  | Ravulizumab | AIFA website | 2020-001497-30 | ALXN1210-COV-305 |
|  | Immunomodulators | Interferon beta-1a | AIFA website | 2020-001366-11 | SOLIDARITY |
|  |  |  |  | 2020-002458-25 | INTERCOP |
|  |  |  |  | 2020-003872-42 | ANTIICIPATE |
|  |  |  |  | 2015-002340-14 | REMA-CAP |
|  |  | CPI-006 | ClinicalTrials.gov | NCT04734873 |  |
|  | Interleukine inhibitors | Sarilumab | AIFA website | 2020-001390-76 | ESCAPE |
|  |  |  |  | 2020-001162-12 | Sarilumab COVID-19 |
|  |  |  |  | 2020-001854-23 | AMMURAVID* |
|  |  |  |  | 2020-001745-40 | COVID-SARI |
|  |  |  |  | 2015-002340-14 | REMAP-CAP |
|  |  | Emapalumab | AIFA website | 2020-001167-93 | Sobi.IMMUNO-101 |
|  |  | Anakinra | AIFA website | 2020-001167-93 | Sobi.IMMUNO-101 |
|  |  |  |  | 2020-005828-11 | SAVE-MORE |
|  |  |  |  | 2015-002340-14 | REMAP-CAP |
|  |  | Tocilizumab | AIFA website | 2020-001110-38 | TOCIVID-19 |
|  |  |  |  | 2020-001386-37 | RCT-TCZ-COVID-19 |
|  |  |  |  | 2020-001154-22 | Tociliz2020-001154-22 |
|  |  |  |  | 2020-001854-23 | AMMURAVID |
|  |  |  |  | 2020-005291-35 | ANTICIPANT Study |
|  |  |  |  | 2015-002340-14 | REMAP-CAP |
|  |  |  | ClinicalTrials.gov | NCT04315480 |  |
|  |  | Canakinumab | AIFA website | 2020-001370-30 | CAN-COVID |
|  |  |  |  | 2020-001854-23 | AMMURAVID |
|  |  | Mavrilimumab | AIFA website | 2020-001795-15 | COMBAT-19 |
|  |  | Siltuximab | AIFA website | 2020-001854-23 | AMMURAVID |
|  |  | Reparixin | AIFA website | 2020-001645-40 | REPAVID-19 |
|  |  |  |  | 2020-005919-51 | REPAVID-19 Phase 3 |
|  | Corticosteroids | Methylprednisolone | AIFA website | 2020-001854-23 | AMMURAVID |
|  |  |  |  | 2020-004323-16 | RCT-MP-COVID-19 |
|  |  |  |  | 2020-001921-30 | STAUNCH |
|  |  |  | ClinicalTrials.gov | NCT04636671 | MEDAS |
| Hormones | Pituitary hormones | Oxytocin | ClinicalTrials.gov | NCT04386447 | OsCOVID19 |
| Statins |  | Simvastatin | AIFA website | 2015-002340-14 | REMAP-CAP |
| Vaccines |  | GRAd-COV2 | AIFA website | 2020-002835-31 | RT-CoV-2 |
|  |  |  |  | 2020-005915-39 | COVITAR |
|  |  | COVID-eVAX | AIFA website | 2020-003734-20 | COVID-eVax |
| Other | | ABX464 | AIFA | 2020-001673-75 | MiR-AGE |
|  |  | Cobicistat | AIFA | 2020-001528-32 | ARCO |
|  |  | DAS181 | ClinicalTrials.gov | NCT04354389 |  |
|  |  | Nafamostat Mesylate | ClinicalTrials.gov | NCT04352400 | RACONA |
|  |  | Pamrevlumab | AIFA | 2020-001472-14 | FibroCov |
|  |  | Vitamin C | AIFA website | 2015-002340-14 | REMAP-CAP |
|  |  |  | ClinicalTrials.gov | NCT04323514 |  |
| Plasma derivatives |  | Convalescent plasma | ClinicalTrials.gov | NCT04385043 | COV2-CP |
|  |  |  |  | NCT04428021 | PLACO-COVID |
|  |  |  |  | NCT04321421 | COV19-PLASMA |
|  |  |  |  | NCT04346589 |  |
|  |  |  |  | NCT04393727 | TSUNAMI |
|  |  |  |  | NCT04418531 |  |
|  |  |  |  | NCT04374526 | LIFESAVER |
|  |  |  |  | NCT04569188 | RESCUE |
|  |  |  |  | NCT04614012 | IMMUNO-COVID19 |
|  |  |  |  | NCT04622826 |  |
|  |  |  |  | NCT04721236 | COV-II-PLA |
|  |  |  |  | NCT04716556 |  |

Classification of drugs/convalescent plasma investigated in COVID-19 interventional clinical trials planned to be carried out in Italy (update: 6 April 2021). § AIFA website (14); ClinicalTrials.gov (16). **#** Study ID is EudraCTR Number for study from AIFA website and NCT Number for study from ClinicalTrials.gov website. * For the study AMMURAVID, the experimental intervention Sarilumab was retrieved from the protocol v3, 17 April 2020.
